# New Acetylenic Norlignan Compounds from Rhizomes of *Curculigo crassifolia*

**DOI:** 10.3390/molecules13081696

**Published:** 2008-08-13

**Authors:** Kai-jin Wang, Ning Li, Hu Wang

**Affiliations:** 1 College of Life Sciences, Anhui University, Hefei 230039, P. R. China; E-mail: wkjahla@sina.com (Kai-jin Wang); 2 Modern Experiment Technology Center, Anhui University, Hefei 230039, P. R. China; E-mail: wanghu@ahu.edu.cn (Hu Wang)

**Keywords:** *Curculigo crassifolia*, Hypoxidaceae, acetylenic norlignan compounds

## Abstract

Two pairs of diastereoisomeric acetylenic norlignan compounds with PhCH(OR_1_)CH(OR_2_)CH_2_C≡CPh skeleta: (1*R*, 2*R*)-1-*O*-methylnyasicoside (**1**) and (1*S*, 2*R*)-1-*O*-methylnyasicoside (**2**), and (1*R*, 2*R*)-crassifogenin D (**3**) and (1*S*, 2*R*)- crassifogenin D (**4**), were isolated from the ethanolic extract of rhizomes of *Curculigo crassifolia*. Compounds **3** and **4** are new and their structures were elucidated on the basis of spectroscopic evidence and comparisons with literature data.

## Introduction

**Figure 1:**
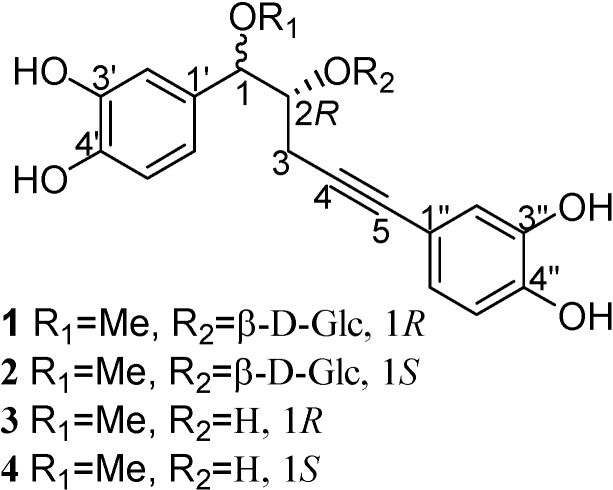
Structures of compounds **1**-**4**.

*Curculigo crassifolia* (Bak.) Hook. f. belongs to the Hypoxidaceae family and is found throughout the Western and Southern regions of China. Its rhizomes are used as a tonic and a folk medicine for treating child pneumonitis [1]. Despite the use of the rhizomes of this plant as a folk remedy, reports on the chemical constituents of this plant are scarce [2,3,4]. In continuation of our studies on the norlignan constituents of the rhizomes of *C. crassifolia*, we now report that this plant is rich in acetylenic norlignan compounds and we describe the isolation and structural elucidation of two pairs of acetylenic norlignans and their corresponding glucosides: (1*R*, 2*R*)-1-*O*-methyl- nyasicoside (**1**) and (1*S*, 2*R*)-1-*O*-methylnyasicoside (**2**), and (1*R*, 2*R*)-crassifogenin D (**3**) and (1*S*, 2*R*)-crassifogenin D (**4**), which contain PhCH(OR_1_)CH(OR_2_)CH_2_C≡CPh moieties (Figure 1). 

## Results and Discussion

The 95% EtOH extract of air-dried and powdered rhizomes of *C. crassifolia* was suspended in H_2_O and then passed through D101 resin column eluting with H_2_O and EtOH. Further repeated column chromatography of the EtOH eluted residue on silica gel and Sephadex LH-20 led to the isolation of two pairs of acetylenic norlignan compounds with PhCH(OR_1_)CH(OR_2_)CH_2_C≡CPh skeletons. Among them, the known compounds **1** and **2** were identified as (1*R*, 2*R*)-1-*O*-methylnyasicoside and (1*S*, 2*R*)-1-*O*-methylnyasicoside by comparing their physical and spectroscopic data with literature values [5,6]. 

**Table 1:** ^1^H-NMR (400 MHz, *δ* in ppm, *J* in Hz) data for compounds **1**-**4** in CD_3_OD.

NO.	1	2	3	4
1	4.38 d (6.28)	4.47 d (3.76)	4.08 d (8.20)	4.09 d (3.40)
2	4.14 m	4.14 m	3.82 m	3.75 m
3	2.70 dd (17.12, 4.76)	2.56 dd (13.84, 5.28)	2.46 dd (16.65, 4.30)	2.46 dd (16.65, 4.30)
	2.30 dd (17.12, 5.20)	2.30 dd (13.84, 4.56)	2.20 dd (16.65, 5.95)	2.20 dd (16.65, 5.95)
2'	6.89 d (1.44)	6.89 d (1.44)	6.87 d (1.50)	6.87 d (1.50)
5'	6.81 d (8.16)	6.81 d (8.16)	6.80 d (8.00)	6.80 d (8.00)
6'	6.75 dd (8.16, 1.44)	6.75 dd (8.16, 1.44)	6.75 dd (8.00, 1.50)	6.75 dd (8.00, 1.50)
2''	6.87 d (1.58)	6.87 d (1.58)	6.82 d (2.00)	6.82 d (2.00)
5''	6.71 d (8.12)	6.71 d (8.12)	6.71 d (8.08)	6.71 d (8.08)
6''	6.80 dd (8.12, 1.58)	6.80 dd (8.12, 1.58)	6.78 dd (8.08, 2.00)	6.78 dd (8.08, 2.00)
OMe	3.25 s	3.37 s	3.18 s	3.18 s
Glc.				
1	4.63 d (7.56)	4.60 d (7.80)		
2	3.30- 3.42 m	3.30- 3.42 m		
3	3.30- 3.42 m	3.30- 3.42 m		
4	3.30- 3.42 m	3.30- 3.42 m		
5	3.30- 3.42 m	3.30- 3.42 m		
6	3.89 dd (11.84, 2.00)	3.89 dd (11.84, 2.00)		
	3.70 dd (11.84, 5.32)	3.70 dd (11.84, 5.32)		

Compound **1**, 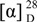
+26.50º (c 0.16, MeOH), was obtained as a white amorphous powder and assigned a molecular formula of C_24_H_28_O_11_ on the basis of the HRFAB-MS (-) (*m/z* 491.1565 [M-1]^-^, calcd. 491.1553). The IR absorption at 3441 cm^-1^ indicated the presence of hydroxyl groups. The ^1^H-NMR spectrum displayed signals for six aromatic protons in two ABX systems, and seven sugar protons, in addition to signals for four aliphatic protons at δ 4.38 (d, H-1), 4.14 (m, H-2), 2.30 (dd, H-3), and 2.70 (dd, H-3). Both sets of ABX systems, one at 6.89 (d, *J* = 1.44 Hz, H-2'), 6.81 (d, *J* = 8.16 Hz, H-5'), and 6.75 (dd, *J* = 8.16, 1.44 Hz, H-6') and the other at 6.87 (d, *J* = 1.58 Hz, H-2''), 6.71 (d, *J* = 8.12 Hz, H-5''), and 6.80 (dd, *J* = 8.12, 1.58 Hz, H-6''), were consistent with two catechol-like moieties, with the latter being conjugated with a acetylene function (*δ* 84.4, 83.7). Analysis of the signals of seven sugar protons suggested a β-D-glucosyl unit with the anomeric proton at *δ* 4.63 (d, *J* = 7.56 Hz). These assignments were made by analyzing the H-H COSY spectrum, incorporating HMQC data. The placement of 1-*O*-methyl and 2-*O*-β-D-Glc was made from the observation of the three-bond coupling of H-1 to C-1 of the methyl group, anomeric proton to C-2, and H-2 to the anomeric carbon in the HMBC spectrum. The two remaining quaternary carbon signals (*δ* 84.4, 83.7) belong to the acetylenic bond. The HMBC spectrum also revealed couplings of H-2 and H-3 to C-4, H-2' and H-6' to C-5. Taking all these chemical shifts and their coupling relationships into consideration, the structure sequence of PhCH(OR_1_)CH(OR_2_)CH_2_C≡CPh for **1** was arrived at, allowing the attachment of a methoxyl group at C-1 position and the β-D-Glc moiety at the C-2 position (Figure 1).

Since compound **1** is a nyasicoside-type norlignan from the *Curculigo* genus, from a biogenetic point of view, the C-2 stereochemistry in **1** should possess a 2*R* configuration [6]. Further comparison of the coupling constant between H-1 and H-2 (6.28 Hz) and the optical rotation (+26.50º) with literature values [5,6], suggest 1*R* and 2*R* stereochemistry in **1**. Hence, **1** is (1*R*, 2*R*)-1-*O*-methyl- nyasicoside.

Compound **2** was obtained as a white amorphous powder and assigned a molecular formula of C_24_H_28_O_11_ from its negative HRFAB-MS data. The ^1^H- and ^13^C-NMR spectra showed that **2** was obtained in a ratio of 1:5 with compound **1**. Most of the NMR signals of the mixture were in pairs. The ^1^H- and ^13^C-NMR spectra of **2** are closely similar to that of **1**, except for this difference of the coupling constant between H-1 and H-2 (*δ* 4.38, d, *J* = 6.28 Hz in **1** and *δ* 4.47, d, *J* = 3.76 Hz in **2**) (Table 1 and Table 2). For instance, **2** displayed signals for two ABX systems belonging to the aromatic protons, protons of a β-D-glucosyl moiety (*δ* 4.60, d, *J* = 7.80 Hz, H-1; *δ* 3.30-3.42, m, H-2-H-5; *δ* 3.89, dd, *J* = 2.00, 11.84 Hz, H-6a; *δ* 3.70, dd, *J* = 5.32, 11.84 Hz, H-6b), and four aliphatic protons at δ 4.47 (d, H-1), 4.14 (m, H-2), 2.30 (dd, H-3), and 2.56 (dd, H-3). These assignments were made by analyzing the H-H COSY spectrum, incorporating HMQC data. The placement of 1-*O*-methyl and 2-*O*-β-Glc was made from the observation of the three-bond coupling of H-1 to methoxy carbon, anomeric proton to C-2, and H-2 to the anomeric carbon in the HMBC spectrum. The two remaining carbon signals (*δ* 84.7, 83.6) belong to the acetylenic bond. These data suggested that **2** and **1** possessed the same norlignan PhCH(OR_1_)CH(OR_2_)CH_2_C≡CPh sequence. From a biogenetic point of view, the configuration of C-2 in **2** should be 2*R* [6]. Further comparing the coupling constant between H-1 and H-2 (3.76 Hz) with literature values [6], this would require 1*S* and 2*R* stereochemistry in **2**. Hence, **2** is (1*S*, 2*R*)-1-*O*- methylnyasicoside.

**Table 2:** ^13^C-NMR (100 MHz, *δ* in ppm) data for compounds **1**-**4** in CD_3_OD^a^.

NO.	1	2	3	4
1	85.8 d	85.6 d	86.4 d	87.0 d
2	79.5 d	79.4 d	73.9 d	74.5 d
3	22.4 t	22.3 t	24.9 t	24.5 t
4	84.4 s	84.7 s	85.6 s	85.2 s
5	83.7 s	83.6 s	82.7 s	82.8 s
1'	130.4 s	130.2 s	131.3 s	131.3 s
2'	116.0 d	116.0 d	115.8 d^a^	115.8 d^a^
3'	145.8 s	145.8 s	145.6 s	145.6 s
4'	146.3 s	146.3 s	145.9 s	145.9 s
5'	116.0 d	116.0 d	115.9 d^a^	115.9 d^a^
6'	120.8 d	120.6 d	120.7 d	120.2 d
1''	116.2 s	116.2 s	116.3 s	116.3 s
2''	119.4 d	119.4 d	119.2 d	119.2 d
3''	146.1 s	146.1 s	145.8 s	145.8 s
4''	146.7 s	146.7 s	146.3 s	146.3 s
5''	116.2 d	116.2 d	116.1 d	116.1 d
6''	124.9 d	124.9 d	124.6 d	124.6 d
OMe	57.1 q	57.3 q	56.8 q	56.8 q
Glc.				
1	102.4 d	102.7 d		
2	74.7 d	74.7 d		
3	77.6 d	77.6 d		
4	71.3 d	71.3 d		
5	77.8 d	77.8 d		
6	62.6 t	62.6 t		

^a^ These values may be interchangeable in the same column.

Although compound **1** was successfully purified, attempts to purify compound **2** failed. Reasons for this could be the small amount present and small differences in the interactions between this pair of diastereoisomers, and the column material used for their separation. Compounds **3** and **4** were assigned to (1*R*, 2*R*)-crassifogenin D (**3**) and (1*S*, 2*R*)-crassifogenin D (**4**); they had the same molecular formula of C_18_H_18_O_6_ on the basis of the HRFAB-MS (-) (*m/z* 329.1037 [M-1]^-^, calcd 329.1025). They were obtained as a 1:1 mixture, unresolvable by TLC and HPLC on account of the small amount obtained (only 4 mg, see Experimental). Most of the NMR signals of the mixture were in pairs. The ^1^H-NMR spectrum showed the presence of two 3,4-disubstituted aromatic rings. According to a selective ^1^H-decoupling experiment, incorporating HMQC and HMBC spectra, compounds **3** and **4** possessed the same norlignan PhCH(OR_1_)CH(OR_2_)CH_2_C≡CPh sequence as compounds **1** and **2**. 1D and 2D NMR spectra showed that compounds **3** and **4** were aglycones of compounds **1** and **2**, respectively. The *δ* values at C-2 in **3** and **4** were shifted upfield 5 - 6 compared to those of **1** and **2**, while the *δ* values at C-1 and C-3 in **3** and **4** were downfield shifted, due to the absence of a β-D-glucose unit at C-2. The *δ* values of remaining carbons in **3** and **4** were similar to the corresponding positions of **1** and **2** (Table 2). The correlation peak between C-1 and protons of OCH_3_ in the HMBC spectra of **3** and **4** confirmed that OCH_3_ was linked at C-1. Compounds **3** and **4** are also nyasicoside-type norlignans, so from a biogenetic point of view, the C-2 stereochemistry in **3** and **4** should possess 2*R* configuration [6]. Further comparing the coupling constant between H-1 and H-2 (8.20 Hz in **3**, and 3.40 Hz in **4**), this would require 1*R* and 2*R* stereochemistry in **3**, and 1*S* and 2*R* stereochemistry in **4**. From the above results and comparison to those of compounds **1** and **2**, the structures of (1*R*, 2*R*)-crassifogenin D (**3**) and (1*S*, 2*R*)-crassifogenin D (**4**) were established as aglycones of compounds **1** and **2**. Compounds **3** and **4** were detected by RP-8 TLC in the EtOH extract, which showed **3** and **4** were not artifacts of **1** and **2** produced by the isolation procedure. Since compounds **3** and **4** were obtained as a 1:1 mixture of (1*R*, 2*R*)-crassifogenin D and (1*S*, 2*R*)-crassifogenin D, the (+)-(1*R*, 2*R*) optical rotation in **3** and the (-)-(1*S*, 2*R*) one in **4** cancel each other out, and a zero optical rotation was observed for the mixture of **3** and **4**. On the other hand, the mixture of **1 ** and **2** was obtained in a ratio of 5:1, so the (+)-(1*R*, 2*R*) configuration in **1** was predominant compared to the (-)-(1*S*, 2*R*) one of the minor component **2**, so an optical rotation of +12.37º was observed for the mixture of **1** and **2**.

## Experimental

### General

The optical rotations were obtained on a JASCO-370 polarimeter. The UV spectra were recorded in MeOH on a UV-2401PC Spectrometer. The IR spectra were recorded on a Bio-Rad FTS-35 spectrometer using KBr pellets. The MS data were obtained on an Autospec-3000 spectrometer operating in negative ion mode. 1D and 2D NMR spectra were measured on a Bruker AM-400 or a Bruker DRX-500 spectrometer with TMS as an internal standard. Column chromatography was performed on Sephadex LH-20 (25-100 µm, Pharmacia Fine Chemical Co. Ltd.) and silica gel (200-300 mesh, Qingdao Haiyang Chemical Co.). TLC was carried on silica gel G precoated plates (Qingdao Haiyang Chemical Co.) and spots were detected by 5% sulfuric acid reagents followed by heating.

### Plant material

The plant material was collected in Eshan Prefecture, Yunnan Province, China, in October 2002 and identified as *Curculigo crassifolia* by Prof. Ping-hua Yu, Kunming Institute of Botany, Chinese Academy of Science, where a voucher specimen (No. 20021018) was deposited.

### Extraction and isolation

The air-dried and powered rhizomes of *C. crassifolia* (10 kg) were extracted with 95% EtOH (3×50 L) at room temperature, then the combined extracts were evaporated *in vacuo* to afford a residue (562 g). The residue was suspended in H_2_O and then passed through D101 resin column eluting with H_2_O and EtOH. The EtOH eluent was concentrated *in vacuo* to give a residue (500 g), which was fractionated by CC (silica gel, 3000 g, 200-300 mesh; with CHCl_3_-MeOH, 9:1) to afford 5 fractions (1-5). Fraction 2 (13 g) was refractionated on a silica gel column (220 g, CHCl_3_-MeOH, 9.5:0.5, 1600 mL) to provide 8 fractions (2-1 to 2-8). Fraction 2-4 (210 mg) was purified by repeated Sephadex LH-20 chromatography (EtOH) to afford a mixture of *(1R, 2R)-crassifogenin D* (**3**) and *(1S, 2R)-crassifogenin D* (**4**) (4 mg): white amorphous powder. 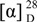
= 0º (*c* 0.12, MeOH); UV (MeOH): λ_max_ (lg *ε*): 205 (4.56), 256 (4.08), 289 (3.77) nm; IR *ν*_max_: 3441, 2924, 1629, 1517, 1443, 1283, 1179, 1111, 815, 583cm^-1^; ^1^H-NMR see Table 1, ^13^C-NMR see Table 2; FAB-MS *m/z*: 329 [M-H]^-^; HR-FAB- MS *m/z*: [M-H]^-^329.1037 (calcd. for C_18_H_17_O_6_, 329.1025). Fraction 5 (210 g) was refractionated by Sephadex LH-20 (EtOH-H_2_O, 0:1-1:0; 2000 mL each eluent) to yield 12 crude fractions (5-1 to 5-12). Fraction 5-7 (4.34 g) was purified by Sephadex LH-20 (EtOH-H_2_O, 0:1-1:0; 700 ml each eluent) to yield 6 fractions (5-7-1 to 5-7-6). Fraction 5-7-4 (612 mg) was repeatedly purified on Sephadex LH-20 (EtOH) to afford a mixture of *(1R, 2R)-1-O-methylnyasicoside* (**1**) and *(1S, 2R)-1-O-methylnyasicoside* (**2**) (212 mg) and pure **1** (18 mg). White amorphous powder; 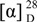: +12.37º (*c* 0.18, MeOH); UV (MeOH): λ_max_ (lg *ε*): 205 (4.34), 255 (3.96), 289 (3.65) nm. IR *ν*_max_: 3441, 2926, 2045, 1546, 1473, 1179cm^-1^; ^1^H-NMR see Table 1; ^13^C-NMR see Table 2; FAB^-^MS *m/z*: 491 [M-H]^-^; HR-FAB-MS *m/z*: [M-H]^- ^491.1565 (calcd. for C_24_H_27_O_11_, 491.1553).
